# Synergistic Antibacterial Effect of Casein-AgNPs Combined with Tigecycline against *Acinetobacter baumannii*

**DOI:** 10.3390/polym13091529

**Published:** 2021-05-10

**Authors:** Yu-Hsuan Chen, Wei-Hsun Wang, Sheng-Hui Lin, Yuan-Ting Yang-Wang, Sung-Pin Tseng, Chi-Sheng Chien, Chi-Jen Shih

**Affiliations:** 1 School of Pharmacy, College of Pharmacy, Kaohsiung Medical University, Kaohsiung 807, Taiwan; ushen80118@gmail.com; 2 Department of Orthopedic Surgery, Changhua Christian Hospital, Changhua 500, Taiwan; wangweihsun@gmail.com; 3 School of Medicine, Kaohsiung Medical University, Kaohsiung 807, Taiwan; 4 Department of Medical Imaging and Radiology, Shu-Zen Junior College of Medicine and Management, Kaohsiung 821, Taiwan; 5 Department of Golden-Ager Industry Management, Chaoyang University of Technology, Taichung 413, Taiwan; 6 Department of Orthopedics, Chi Mei Medical Center, Tainan 710, Taiwan; danteslin@gmail.com; 7 Department of Leisure and Sports Management, Far East University, Tainan 744, Taiwan; 8 Department of Fragrance and Cosmetic Science, College of Pharmacy, Kaohsiung Medical University, Kaohsiung 807, Taiwan; kmu102012062@gmail.com; 9 Department of Medical Laboratory Science and Biotechnology, College of Health Science, Kaohsiung Medical University, Kaohsiung 807, Taiwan; tsengsp@kmu.edu.tw; 10 Department of Electrical Engineering, Southern Taiwan University of Science and Technology, Tainan 710, Taiwan; 11 Department of Medical Research, Kaohsiung Medical University Hospital, Kaohsiung 807, Taiwan; 12 Drug Development and Value Creation Research Center, Kaohsiung Medical University, Kaohsiung 807, Taiwan

**Keywords:** synergistic antibacterial effect, AgNP, tigecycline, *A. baumannii*, silver protein

## Abstract

*Acinetobacter baumannii* (*A. baumannii*) is a common and challenging pathogen of nosocomial infections, due to its ability to survive on inanimate objects, desiccation tolerance, and resistance to disinfectants. In this study, we investigated an antibacterial strategy to combat *A. baumannii* via the combination of antibiotics and silver protein. This strategy used a functional platform consisting of silver nanoparticles (AgNPs) resurrected from silver-based calcium thiophosphate (SSCP) through casein and arginine. Then, the silver protein was combined with tigecycline, the first drug in glycylcycline antibiotic, to synergistically inhibit the viability of *A. baumannii*. The synergistic antibacterial activity was confirmed by the 96-well checkerboard method to determine their minimum inhibitory concentrations (MIC) and calculated for the combination index (CI). The MIC of the combination of silver protein and tigecycline (0.31 mg/mL, 0.16 µg/mL) was significantly lower than that of the individual MIC, and the CI was 0.59, which indicates a synergistic effect. Consequently, we integrated the detailed synergistic antibacterial properties when silver protein was combined with tigecycline. The result could make for a promising approach for the treatment of *A. baumannii*.

## 1. Introduction

Infections from common pathogens, such as *Staphylococcus aureus*, *Pseudomonas aeruginosa* and *Acinetobacter baumannii*, have resulted in difficulties [1,2]. Infectious patients may extend their inpatient days, require more medical resources, and increase the economic burden [3]. Among such pathogens, *A. baumannii* is mostly acquired within health-care facilities. Due to its ability to survive on inanimate objects, desiccation tolerance, and resistance to disinfectants, *A. baumannii* has become a global medical challenge [4,5]. However, the available antibiotics are now less efficacious, and the options for treatment are limited [6,7].

Tigecycline is considered to be a last-resort antibiotic with broad-spectrum activities against many Gram-positive bacteria, Gram-negative bacteria, anaerobes, and even drug-resistant pathogens [8,9]. The dosage of tigecycline has been increasing because of the higher incidence of infection that does not respond to first-line antibiotics. Tigecycline consists of a glycylcycline structure that interacts with the bacterial 30S ribosome subunit to inhibit bacterial protein synthesis. Presently, to combat *A. baumannii* infection, a practical therapy has been used combining tigecycline and other antibiotics, such as colistin, amikacin, levofloxacin, rifampicin, and so on [6].

However, combination therapies may cause several problems of overuse and abuse of antibiotics, and we propose the AgNPs combining with tigecycline as alternative antibacterial agents [8,10]. Therefore, a current antibacterial strategy is to develop a combination of antibacterial materials and antibiotics to combat pathogens. Metallic ions and metal nanoparticles are considered therapeutic agents, such as Ag^+^, Cu^2+^, Cu^+^, and Au^2+^ [11,12]. Among these, silver nanoparticles (AgNPs) are well known for their outstanding broad-spectrum antibacterial ability. In terms of the antibacterial mechanism, AgNPs contact the bacterial membrane and then damage the cell wall. AgNPs and Ag ions entering bacterial cells may increase reactive oxygen species (ROS) production and block the electron transport chain and eventually inhibit the synthesis of ATP [13]. However, the agglomeration of AgNPs would lose their nanosized characteristics and decrease antibacterial ability.

The green synthesis of nanoparticles has been considered to be a cost-effective, environmentally friendly, and lower-toxicity method compared to other chemical synthesis methods. Previous studies have investigated enzymes [14], bacteria [14], proteins [15], and plant extracts [16,17,18] as green precursors to prepare nanoparticles. There are some bio-based resources to maintain stabilized AgNPs reagent, such as plant extracts [16,17,18,19,20,21] and protein. Various proteins have been successfully used as carriers with advantages of preventing chemical degradation, increasing bioavailability, and controlling delivery [16,22,23,24]. Here, we investigated casein presenting a stabilizing effect on the AgNPs, and arginine possessing high affinity towards silver ions.

Therefore, AgNPs might be a potent antibacterial agent in combination with antibiotics for enhancing synergistic effects [25,26]. Pajares-Chamorro et al. demonstrated that silver-containing materials combined with antibiotics showed antibacterial efficacy through electron microscopy observation [27]. However, the bacterial growth kinetics in the 24 h following application of this combination have been less well investigated in previous works and did not demonstrate a combinatorial relationship. Tigecycline has been considered a last-line antibiotic against drug-resistant bacteria [28]. Moreover, the synergistic antibacterial activity of tigecycline combined with casein-AgNPs has yet to be studied.

In this study, we focused on the combination of silver protein with tigecycline and their synergistic effect against *A. baumannii*. AgNPs were based on casein from silver-containing silica-based calcium phosphate (SSCP) with a 1% Ag molar ratio. Casein and arginine inspire AgNPs from SSCP as green synthesis. Casein could inspire silver nanoparticles to release from SSCP and stabilize as colloid. Then, arginine combining with casein-AgNPs increase the release of silver, because arginine has the highest affinity towards silver ions. To characterize the morphology of AgNPs, they were confirmed by TEM images and UV-visible spectroscopy. The size and distribution of the AgNPs were measured with at least 30 particles to obtain representative data. Furthermore, casein-AgNPs combined with tigecycline was investigated as a potent candidate for infection treatment. To evaluate the synergistic antibacterial efficacy of this combination, the checkerboard method was used to obtain their combination index (CI) values. Then, interactions related to time-kill kinetic curves and colony-forming assays were used to determine the minimum inhibition concentration (MIC) and the minimum bactericidal concentration (MBC). Furthermore, we proposed an ecofriendly approach to prepare casein-AgNPs with biocompatibility and antibacterial ability to combat infectious pathogen. These multiple properties will be suitable to apply as antibacterial coatings for medical devices. Furthermore, we proposed the casein-AgNPs with biocompatibility and antibacterial ability to combat infectious pathogens. The casein-AgNPs would be the suitable application immobilized on nylon and silk fibers. These modified polymeric materials with antibacterial properties may have potential to be applied as antibacterial textile [18]. Moreover, the casein-AgNPs are also suitable for application as antibacterial coatings for medical devices, such as urinary catheters and nasogastric tubes.

## 2. Materials and Methods

### 2.1. Materials

Pluronic F-127 was obtained from BASF Inc. (Ludwigshafen am Rhein, Germany), TEOS (98.0% purity) from ACROS Organics (Trenton, NJ, USA), calcium nitrate tetrahydrate (98.5% purity) from SHOWA (Ibaraki Prefecture, Japan), triethyl phosphate (98.0% purity) from FLUKA (Asheville, NC, USA), silver nitrate (99.8% purity) from SHOWA (Ibaraki Prefecture, Japan) and nitric acid (99.5% purity) from SHOWA (Ibaraki Prefecture, Japan). Tigecycline (Tygacil^®^) was purchased from Wyeth Pharmaceuticals Inc. (Collegeville, PA, USA). *A. baumannii* (ATCC BAA-747) was used for the antibacterial assay, was provided by the American Type Culture Collection (Manassas, VA, USA).

### 2.2. Synthesis of the SSCP

The mesoporous bioactive glass comprising 80SiO_2_-15CaO-5P_2_O_5_-1Ag_2_O was synthesized by the EISA (evaporation-induced self-assembly) method [19,20]. During the synthesis, 7 g non-surfactant Pluronic F127 (BASF, Ludwigshafen am Rhein, Germany) was used as the structure-directing agent, and the precursors including 6.7 g tetraethyl orthosilicate, TEOS (ACROS, Trenton, NJ, USA), 0.73 g triethyl phosphate, TEP (FLUKA, Asheville, NC, USA), 1.4 g calcium nitratetetrahydrate, Ca (NO_3_)_2_.4H_2_O (SHOWA, Ibaraki Prefecture, Japan) and 0.13 g silver nitrate (AgNO_3_, SHOWA, Ibaraki Prefecture, Japan) were used. The above substances were dissolved in ethanol with 2M HNO_3_ (SHOWA, Ibaraki Prefecture, Japan) and stirred at room temperature for 24 h to form a sol. We further immersed polyurethane foams (PUF) in the sol, which was then subjected to compression and release twice. Then, the PUF-coated sol was dried at 100 °C for 24 h as the aging process. Afterwards, the PUF was heated for 2 h at 600 °C with a constant heating rate of 10 °C/min to remove the surfactants. Consequently, the SSCP powders were produced.

### 2.3. Synthesis of Casein/Arginine-AgNPs Reagent

The solid compositions of the SSCP powders immersed with 20 mg/mL Tryptic Soy Broth medium (containing casein and arginine solution, as a ratio 1:1) at 150 rpm for 24 h. Then, The SSCP powder were extracted by 0.22 µm syringe filter and the suspension was collected as casein/arginine-AgNPs.

### 2.4. Characterization of Casein/Arginine-AgNPs

The casein/arginine-AgNPs reagents were measured by a DU730 UV-visible spectrometer (Beckman coulter, Brea, CA, USA) and the absorbance recorded in a 200–700 nm range of wavelengths. To characterize the structure morphology of casein/arginine-AgNP reagents, we obtained transmission electron microscopy (TEM) images to observe its dispersed solution using a JEM-2100 transmission electron microscope (JEOL, Tokyo, Japan) equipped with a LaB_6_ gun at 120 kV. The size and size distribution of the AgNPs were measured with at least 30 particles to obtain representative data.

### 2.5. In Vitro Antibacterial Test

#### 2.5.1. Bacteria Culture and Conservation

The tested strain, *Acinetobacter baumannii* Bouvet and Grimont (ATCC^®^ BAA-747™) was obtained from the American Type Culture Collection (ATCC) (Manassas, VA, USA). According to the Bioresource Collection and Research Center (BCRC), Hsinchu, Taiwan instructions, to revive the freeze-dried *A. baumannii*, we used 6 mL trypticase soy broth (TSB) medium to rehydrate the pellet, and then mix the entire suspension. Then, 10 µL suspension was dropped on trypticase soy sgar (TSA) to cultivate at 37 °C for 24 h in an oxygenic environment. After 24 h, by the streak-plate method, the forming colony on TSA plate would move to TSB medium and mixed with glycerol stock to preserve at −80 °C freezer for conservation the bacteria.

#### 2.5.2. Time-Kill Kinetic Assay

##### Determination of Minimal Inhibitory Concentration (MIC)

A time-kill kinetic assay was carried out using the microtiter plate method as previously described. Two treatment samples (casein/arginine-AgNPs and tigecycline) were measured against *A. baumannii*. A 10 µL bacterial suspension was added to the corresponding wells of a 96-flat-bottom-well microtiter plate at a final concentration of 5 × 10^5^ to 10^6^ CFU/mL (from a 0.5 McFarland (≈10^8^ CFU/mL) stock) and the corresponding concentrations of either 100 µL of casein/arginine-AgNPs (0.16–20 mg/mL in 2-fold dilutions) or 100 µL of tigecycline (0.16–10 µg/mL in 2-fold dilutions) in a final volume of 210 µL per well. Then, we used a spectrophotometer (Tecan, Mannedorf, Switzerland) to observe the bacterial growth kinetics of the optical density at 600 nm every hour (up to 24 h) at 37 °C. Every interval before testing, the plate would be shaken for 3 s. The plates were sealed during the whole experiment. A sterilized condensation water was used in the incubator to maintain humidity. The MIC was identified as the minimum concentration that showed no visible growth after 24 h. The measurements were performed against *A. baumannii* in triplicates.

##### Determination of Minimal Bactericidal Concentration (MBC)

Determination of MBC was measured by colony counting of the cultures corresponding to no visible growth in the microdilution experiments described above. In the testing process, the suspension tested by the above microdilution measurement, would then streak 100 µL suspension to fresh TSA by sterile swabs and incubated at 37 °C for an additional 24 h. MBC was considered to be the minimal concentration of antibacterial agent that reduces initial concentration of bacteria in the culture to less than 0.1%, and did not form a single bacterial colony on the TSA medium.

##### Checkerboard Assay

The impact on the combination of casein/arginine-AgNPs and tigecycline was determined by broth microdilution assay in checkerboard testing as previously described [29]. The combination of casein/arginine-AgNPs and tigecycline diluted 2-fold in a 96-well microplate. The solutions containing 100 µL of casein/arginine-AgNPs (0.16–20 mg/mL in 2-fold dilutions) in the raw and 100 µL of tigecycline (0.16–10 µg/mL in 2-fold dilutions) in a final volume of 200 µL per well. The *A. baumannii* bacteria were suspended in tryptic soy broth (TSB) medium and adjusted to 0.5 McFarland suspension, which corresponds to 1 × 10^8^ colony-forming units/mL (CFU/mL). Then, the *A. baumannii* suspension was added to the combination solution to a final concentration of 5 × 10^5^ CFU/mL and cultured at 37 °C for 24 h. The MIC was defined as the minimum concentration that resulted in no visible growth after 24 h. The optical density at 600 nm of the solutions was measured to confirm their MIC using a spectrophotometer (Tecan, Mannedorf, Switzerland). All experiments were done in triplicates. Furthermore, the interaction of two antibacterial agents was analyzed by the combination index (CI), according to Chou et al. [30], using the following equation:

CI =(D1/Df_1_) + (D2/Df_2_) + (D1 × D2/Df_1_ × Df_2_) (1)

where D1 and D2 are the MICs of each antibacterial agent in combination (in a single well), and Df_1_ and Df_2_ are the MICs of each antibacterial agent individually. The CI values of two antibacterial agents were defined as synergism (CI < 1), additive effect (CI = 1) and antagonism (CI > 1) [31].

### 2.6. Statistical Analysis of Data

Experiment data were presented as mean ± standard deviation. Results were statistically compared by the one-way Analysis of Variance (ANOVA) test. The statistical difference was considered to be significant when the *p* < 0.05.

## 3. Results and Discussion

### 3.1. Nanomaterial Characteristics

**Figure 1:**
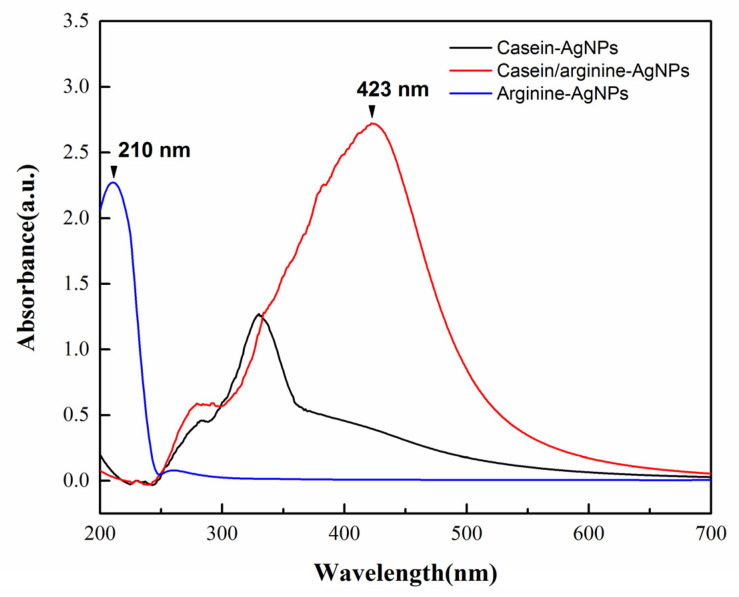
UV-visible spectra of casein, casein/arginine-AgNPs, and arginine-AgNPs.

In this study, we proposed a new colloid system, casein-AgNPs (colloid type), based on our previous work, SSCP (powder type) [29,32]. Different forms of silver between casein-AgNPs, casein/arginine-AgNPs, and arginine-AgNPs are shown in Figure 1. The spectra of arginine-AgNPs, the absorption bands located between 200 and 230 nm for materials containing Ag_2_O, is due to the 4d^10^ to 4d^9^5s^1^ transition of the Ag^+^ ions [22]. In contrast, the spectra of casein/arginine-AgNPs appeared a peak at 423 nm which can be assigned to silver nanoparticles. Furthermore, the spectrum of arginine presented more silver ions than larger silver nanoparticles. T Arginine has the highest affinity towards silver ions among all amino acids, which may bind at various electron rich sites, e.g., nitrogen atoms of α-amino groups as well as guanidino side chains, in addition to carboxyl moieties at the C-terminus [33,34]. The use of casein proteins is considered to produce bio-tolerable and highly stable silver nanoparticles with a fair control over their size without using any additional reducing agent [15,35]. As a result, casein/arginine-AgNPs would form stable silver–arginine complexes.

**Figure 2:**
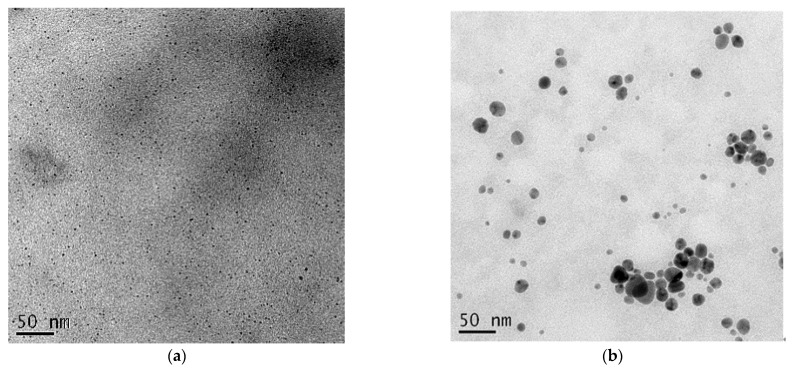

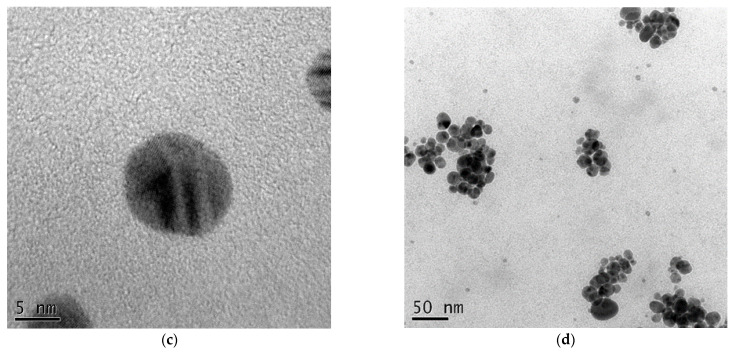
TEM images of (**a**) Casein-AgNPs; (**b**,**c**) Casein/arginine-AgNPs and (**d**) Casein/arginine-AgNPs (after 3 months).

To characterize the morphology of AgNPs of casein-AgNPs, they were confirmed by TEM images shown in Figure 2. The size and size distribution of the AgNPs were measured with at least 30 particles to obtain representative data, shown in Table 1. In Figure 2a, the TEM images revealed casein-AgNPs dispersed homogeneously, and the AgNPs presented average size of 2.9 nm. In contrast, in Figure 2b, casein/arginine-AgNPs have larger spherical sizes ranging from 10.3 to 24.2 nm, and the average size was 17.0 nm and no aggregation of AgNPs was observed inside the casein matrix. Moreover, in Figure 2c, casein/arginin-AgNPs revealed the polycrystalline structure of AgNPs. The TEM images were in close agreement with the spectra obtained using UV-visible spectroscopy, indicating the formation of fairly uniform silver nanoparticles under optimum conditions. Moreover, the TEM image and average particle sizes of casein/arginine-AgNPs (after 3 months) are presented in Figure 2d and Table 1 to confirm the stability of synthesized AgNPs. The casein/arginine-AgNPs dispersed as spheres homogeneously and their particle sizes ranged from 8.6 to 30.8 nm and the average size was 17.2 nm using one-way ANOVA statistical analysis. There is no significant difference between AgNPs synthesized after three months.

In TEM images, we observed casein-AgNPs of average size 2.9 nm and casein/arginine-AgNPs of average size 17.0 nm. To explain the AgNPs formation process, the model of recognition–reduction–limited nucleation was used [36]. Moreover, proteins inspired silver ions from SSCP matrix. Then, the silver nuclei and proteins combined, causing growth by more reduction of silver ions and accumulation on these nuclei. Additionally, the protein linkage and lots of biomolecules suspended in the reaction solutions would improve stable spherical AgNPs formation. With sufficient aging time, large-sized AgNPs were obtained, and the crystalline phase transferred from polycrystalline to single crystalline by Ostwald ripening. Casein presented a stabilizing effect on the AgNPs, and arginine possessed high affinity towards metals for larger particle formation.

### 3.2. Antibacterial Activity of Casein/Arginine-AgNPs

**Figure 3:**
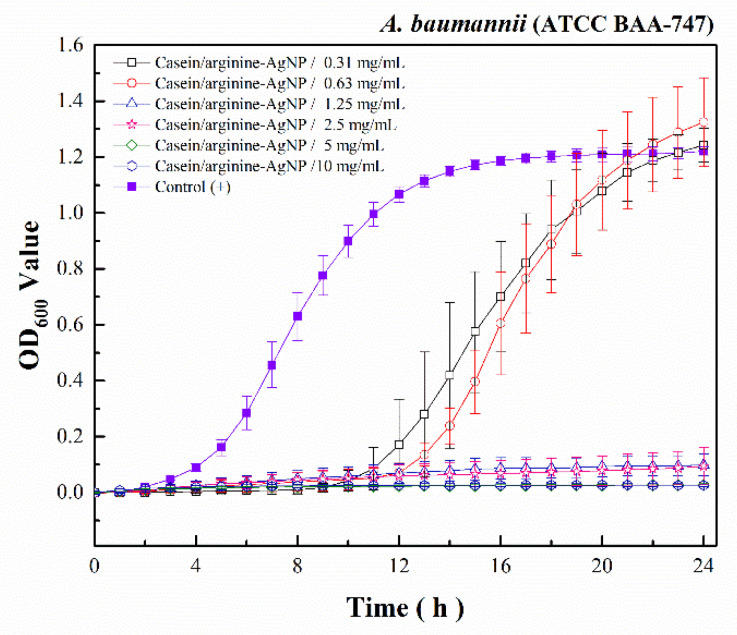
The *A. baumannii* growth kinetic results of casein/arginine-AgNPs.

**Figure 4:**
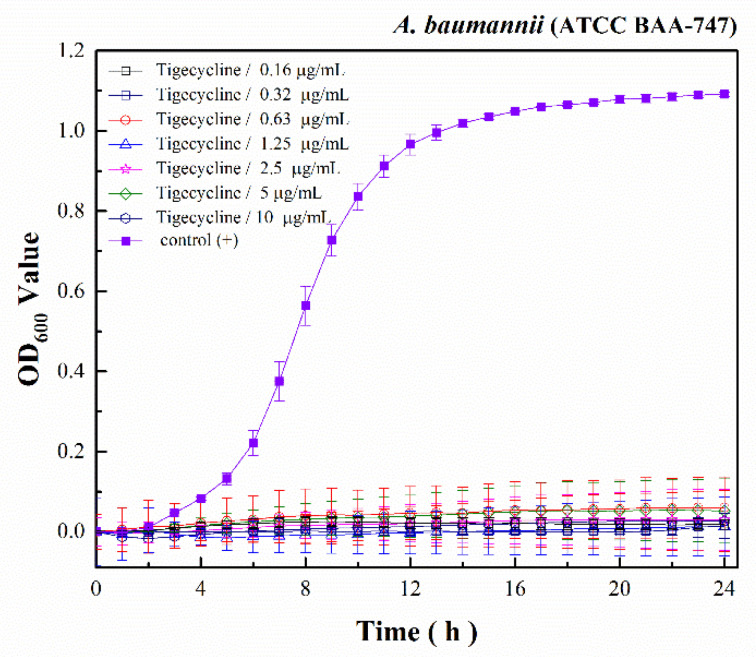
The *A. baumannii* growth kinetics of tigecycline.

Antibacterial effects depend on the composition, size, shape, and synthesis method of each AgNPs. In our previous study, we provided the mesoporous SSCP to restrict AgNPs in small sizes to avoid agglomeration. Then, we used casein to inspire AgNP from SSCP and stabilize AgNPs. To examine the antimicrobial activity of casein/arginine-AgNPs, alone and in combination with tigecycline, antibacterial susceptibility measurements were conducted against *A. baumannii*. As shown in Figure 3, casein/arginine-AgNPs showed antibacterial activity against *A. baumannii* with MIC values of 1.25 mg/mL. For the *A. baumannii* growth kinetics, the treatment with casein/arginine-AgNPs after 10 h, less than 0.63 mg/mL, and viable bacterial growth was observed. According to the colony-forming results in Figure 4, the MBC value of casein/arginine-AgNPs is 1.25 mg/mL, which agrees with the MIC.

As shown in Figure 3, casein/arginine-AgNPs showed antibacterial activity against *A. baumannii* with MIC values of 1.25 mg/mL. For the time-kill kinetic curves, after 10 h of treatment with casein/arginine-AgNPs less than 0.63 mg/mL, viable bacterial growth was observed. Moreover, AgNPs were more effective against Gram-negative bacteria than against Gram-positive bacteria, as previously described, due to the membrane layer of the bacterial structure.

Tigecycline showed antibacterial ability against *A. baumannii* with MIC values of 2.5 µg/mL and finish with Figure 4. Moreover, the 2 MIC (5 µg/mL) and 4 MIC (10 µg/mL) growth kinetics of tigecycline represented a slight increase in optical density (OD) values at a wavelength of 600 nm. In contrast, the 1/2 MIC (0.63 µg/mL) and 1/4 MIC (0.31 µg/mL) growth kinetics of casein/arginine-AgNPs experienced a significant increase.

### 3.3. Synergy Effect of the Combination of Tigecycline and Casein/Arginine-AgNPs

The results of the checkerboard assay confirmed that there was a synergistic antibacterial effect between AgNPs and tigecycline, as shown in Table 2. According to the lowest CI values (CI = 0.59), the result showed the best synergistic effect with casein/arginine-AgNPs at 1/2 MIC (0.31 mg/mL) and tigecycline at 1/16 (0.16 µg/mL). The synergistic effect was also present with the combination of casein/arginine-AgNPs at 1/2 MIC (0.31 mg/mL) and tigecycline at 1/4 and 1/8 MIC (0.63 and 0.31 µg/mL). Moreover, the combination of casein/arginine-AgNPs at 1/4 MIC (0.16 mg/mL) and tigecycline at 1/2 MIC (1.25 µg/mL) showed the synergistic effect as well.

**Figure 5:**
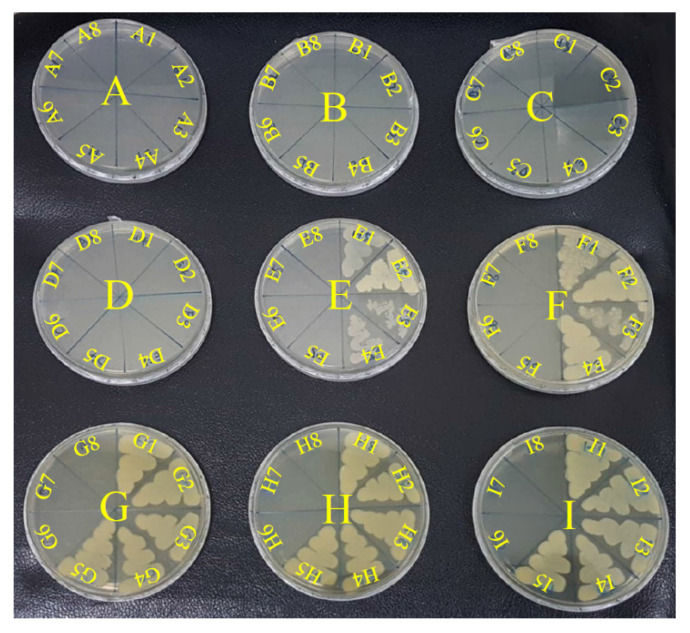
The colony-forming capacity results of the combination of casein/arginine-AgNPs and tigecycline. The combination of A series (casein/arginine-AgNPs = 10 mg/mL; A1–A8 Tigecycline = 0, 0.16, 0.31, 0.63, 1.25, 2.5, 5, 10 µg/mL); B series (casein/arginine-AgNPs = 5 mg/mL; B1–B8 Tigecycline = 0, 0.16, 0.31, 0.63, 1.25, 2.5, 5, 10 µg/mL); C series (casein/arginine-AgNPs = 2.5 mg/mL; C1–C8 Tigecycline = 0, 0.16, 0.31, 0.63, 1.25, 2.5, 5, 10 µg/mL); D series (casein/arginine-AgNPs = 1.25 mg/mL; D1–D8 Tigecycline = 0, 0.16, 0.31, 0.63, 1.25, 2.5, 5, 10 µg/mL); E series (casein/arginine-AgNPs = 0.63 mg/mL; E1–E8 Tigecycline = 0, 0.16, 0.31, 0.63, 1.25, 2.5, 5, 10 µg/mL); F series (casein/arginine-AgNPs = 0.31 mg/mL; F1–F8 Tigecycline = 0, 0.16, 0.31, 0.63, 1.25, 2.5, 5, 10 µg/mL); G series (casein/arginine-AgNPs = 0.16 mg/mL; G1–G8 Tigecycline = 0, 0.16, 0.31, 0.63, 1.25, 2.5, 5, 10 µg/mL); H series (casein/arginine-AgNPs = 0.08 mg/mL; H1–H8 Tigecycline = 0, 0.16, 0.31, 0.63, 1.25, 2.5, 5, 10 µg/mL); I series (casein/arginine-AgNPs = 0 mg/mL; I1–I8 Tigecycline = 0, 0.16, 0.31, 0.63, 1.25, 2.5, 5, 10 µg/mL).

Furthermore, as a result of the colony-forming assay in Figure 5, the synergistic combination provided their bactericidal ability with culturing for an additional 24 h. There is little attention paid to investigating the synergistic combination with silver to combat *A. baumannii*. First, the colony-forming results of casein/arginine-AgNPs with the concentration of 10, 5, 2.5, 1.25, 0.63, 0.31, 0.16, and 0.08 mg/mL are presented in A1, B1, C1, D1, E1, F1, G1, and H1. We observed a small number of bacteria in 0.63 mg/mL (E1) and 0.31 mg/mL (F1), indicating gradual bacterial inhibition. This is caused by the antibacterial mechanism of silver ions decreasing bacteria proliferation and increasing apoptosis in *A. baumannii* [32].

On the other hand, the bactericidal results of tigecycline alone with concentrations of 10, 5, 2.5, 1.25, 0.63, 0.31, and 0.16 µg/mL are shown in I2, I3, I4, I5, I6, I7, and I8. The bacterial colony results show complete inhibition, rather than gradual inhibition. In this study, we integrated the effects of AgNPs and tigecycline by determining bacterial growth kinetics at 24 h and performing a colony-forming assay at 48 h. However, the combinations for bacterial colonization assay were less effective to kill *A. baumannii* completely, due to its Gram-negative structure. The Gram-negative bacteria is composed of a thin peptidoglycan layer sandwiched between an inner cytoplasmic cell membrane and a bacterial outer membrane [37].

As a result, we integrated the synergistic antibacterial ability of casein/arginine-AgNPs at 1/2 MIC (0.31 mg/mL) and tigecycline at 1/16 (0.16 µg/mL) in combination. We proposed the combination of AgNP and tigecycline providing two different antibacterial mechanisms to increase its efficiency. AgNPs and tigecycline also showed different degrees of antibacterial activity in relation to their material properties [37]. First, AgNPs would be oxidized as partial oxidation to form Ag_2_O and then release silver ions [38]. Silver ions would be incorporated into bacterial cell membranes and bind to membrane proteins to block their respiratory system [39]. On the other hand, the antibacterial mechanism of tigecycline is due to its glycylcycline structure interacting with the bacterial 30S ribosome subunit, blocking the entry of transfer RNA to block protein synthesis. Meanwhile, chloramphenicol and kanamycin also show a similar mechanism of inhibiting bacterial protein synthesis. Pajares-Chamorro et al. provided the synergistic effect of similar silver-containing materials combined with antibiotics, although their exact combinatorial relationship was not extensively discussed. In other previous studies, a synergistic effect was shown as AgNPs combining with common antibiotics, including gentamycin, amoxicillin, linezolid and vancomycin, against pathogens [25,39].

Based on the results of synergistic antibacterial ability, the combination of casein-AgNPs and tigecycline could suppress the growth of *A. baumannii*, which should be further confirmed in animal models and clinical trials. Moreover, we supposed the ecofriendly approach to prepare AgNPs with efficient antibacterial ability would be suitably applied as antibacterial fibers such as wound dressing and antibacterial coatings for medical devices, such as urinary catheters and nasogastric tubes.

## 4. Conclusions

In this study, we investigated the combination of casein/arginine-AgNPs with tigecycline as a new formulation to combat *A. baumannii*. For antibacterial material, casein and arginine would increase nucleation of AgNPs from SSCP. The average size of AgNPs in 17.0 nm inside the casein protein was observed by TEM image. In an in vitro antibacterial test, the MRSA time-kill kinetics showed the MIC of casein/arginine-AgNPs with 1.25 mg/mL. Moreover, the combination composed with casein/arginine-AgNPs at 1/2 MIC (0.31 mg/mL) and tigecycline at 1/16 (0.16 µg/mL) presented the CI value (0.59) for synergistic antibacterial ability by a checkerboard method. Consequently, based on the results of synergistic antibacterial ability, the combination of casein-AgNPs and tigecycline could suppress the growth of *A. baumannii*, which should be further confirmed in animal models and clinical trials. Moreover, we suppose the materials as antibacterial agent could be applied as antibacterial coatings for medical devices.

## Figures and Tables

**Table 1:** Size distribution of AgNPs (n = 30). (*p* < 0.05)

Silver Reagent	Size Distribution of AgNPs (nm)	Average Size of AgNPs (nm)
Casein-AgNPs	1.7–4.6	2.9 ± 0.8
Casein/arginine-AgNPs	10.3–24.2	17.0 ± 4.5
Casein/arginine-AgNPs (after 3 months)	8.6–30.8	17.2 ± 4.2

**Table 2:** MIC of different antibacterial agents for *A. baumannii* and the calculated CI values.

Sample Name	MIC of Tigecycline (µg/mL)	MIC of Casein/Arginine-AgNPs (mg/mL)	CI
Casein/arginine-AgNPs and Tigecycline	0.16	1.25	0.88
	0.31	0.63	0.88
	0.31	0.31	0.69
	0.31	0.16	0.59

## Data Availability

The data presented in this study are available on request from the corresponding author.
